# Host Long Noncoding RNA lncRNA-PAAN Regulates the Replication of Influenza A Virus

**DOI:** 10.3390/v10060330

**Published:** 2018-06-16

**Authors:** Jing Wang, Yujia Wang, Rui Zhou, Jianyuan Zhao, Yongxin Zhang, Dongrong Yi, Quanjie Li, Jinming Zhou, Fei Guo, Chen Liang, Xiaoyu Li, Shan Cen

**Affiliations:** 1 Institute of Medicinal Biotechnology, Chinese Academy of Medical Sciences and Peking Union Medical School, Beijing 100050, China; jingwang@imb.pumc.edu.cn (J.W.); yujia.wang@imb.pumc.edu.cn (Y.W.); ruizhou12345@126.com (R.Z.); zjyuan815@163.com (J.Z.); yongxinzhang@imb.pumc.edu.cn (Y.Z.); dongrong.yi@imb.pumc.edu.cn (D.Y.); quanjie.li@imb.pumc.edu.cn (Q.L.); zhoujinming@imb.pumc.edu.cn (J.Z.); lixiaoyu@imb.pumc.edu.cn (X.L.); 2 Institute of Pathogen Biology, Chinese Academy of Medical Sciences and Peking Union Medical School, Beijing 100730, China; guoafei@ipbcams.ac.cn; 3 Lady Davis Institute for Medical Research, Jewish General Hospital, McGill University, Montreal, QC H3T 1E2, Canada; chen.liang@mcgill.ca

**Keywords:** lncRNA, influenza A virus, PAAN, polymerase basic protein 1 (PB1), polymerase acidic protein (PA), RdRp

## Abstract

The productive infection of influenza A virus (IAV) depends on host factors. However, the involvement of long non-coding RNAs (lncRNAs) in IAV infection remains largely uninvestigated. In this work, we have discovered a human lncRNA, named lncRNA-PAAN (PA-associated noncoding RNA) that enhances IAV replication. The level of lncRNA-PAAN increases upon infection of IAV, but not other viruses, nor interferon treatment, suggesting specific up-regulation of lncRNA-PAAN expression by IAV. Silencing lncRNA-PAAN significantly decreases IAV replication through impairing the activity of viral RNA-dependent RNA polymerase (RdRp). This function of lncRNA-PAAN is a result of its association with viral PA protein, a key component of IAV RNA polymerase complex. Consequently, depletion of lncRNA-PAAN prevents the formation of functional RdRp. Together, these results suggest that lncRNA-PAAN promotes the assembly of viral RNA polymerase, thus warranting efficient viral RNA synthesis. Elucidating the functions of lncRNAs in IAV infection is expected to advance our understanding of IAV pathogenesis and open new avenues to the development of novel anti-IAV therapeutics.

## 1. Introduction

Influenza A virus (IAV) infection causes serious respiratory disease and mortality. In addition to annual epidemics, IAV has caused multiple pandemics over the past century, thus presents great threat to public health and leads to high economic loss [1,2,3]. IAV belongs to *Orthomyxoviridae*, contains eight segments of single-stranded negative-sense RNA (vRNA) that together encode more than 10 viral proteins including several splice variants. Each vRNA is coated with viral nucleoprotein (NP), its 5′ and 3′ ends are bound by a single copy of viral RNA-dependent RNA polymerase (RdRp) complex. RdRp is composed of PB1, PB2 and PA subunits, together with the NP, catalyzes viral RNA transcription (vRNA to mRNA) and replication (vRNA to cRNA to vRNA) [4,5,6]. IAV infection begins with the viral hemagglutinin (HA) protein binding to the sailic acid at the surface of host cell, followed virion internalization and fusion of cellular and viral membranes. Viral ribonucleoprotein (vRNP) is then released into the cytosol, and further imported into the nucleus where transcription and replication of the viral genome occur. In the late stage of IAV replication, the newly assembled vRNP together with M1, M2, HA and neuraminidase (NA) generate the budding virions which are released from the host cell surface with the help of NA [7,8,9].

Successful IAV infection requires the active involvement of many host factors. In addition to proteins and microRNAs, long non-coding RNAs (lncRNAs) have also been reported to participate in viral replication [10,11,12]. lncRNAs are transcripts longer than 200 nucleotides but lack significant protein-coding capacity. Most of them are synthesized by RNA polymerase II, undergo splicing. Their expression is often tissue specific and occurs at specific developmental stages [13,14,15]. lncRNAs regulate various biological processes, including cell growth and differentiation, apoptosis, immune responses, and tumorigenesis [16,17,18,19]. In addition, expression of lncRNAs changes in response to viral infections, including IAV infection of human lung epithelial cells [20,21,22]. Not surprisingly, lncRNAs have been reported to modulate viral infection by different mechanisms. For instance, virus-induced lncRNA-ACOD1 (aconitate decarboxylase 1) binds to the glutamic-oxaloacetic transaminase (GOT2) and facilitates viral replication through simulating GOT2 activity and its metabolites [23]. Another lncRNA NRAV (negative regulator of antiviral response) was shown to significantly promote IAV replication through suppressing transcription of interferon-stimulated genes, which highlights the role of lncRNAs in modulating host antiviral response [21]. These few examples mark the beginning of our efforts to understand the potentially diverse functions of lncRNA in modulating viral infection.

## 2. Materials and Methods

### 2.1. Plasmids and Antibodies

pHW181-PB2, pHW182-PB1, pHW183-PA, pHW185-NP and PolI-luc plasmids are derived from influenza A/WSN/33 virus [24]. Rabbit anti-PB1, anti-PB2, anti-PA, anti-NP, and mouse anti-M1, anti-GAPDH, anti-p84 antibodies were purchased from GeneTex (Irvine, CA, USA). Mouse anti-β-actin antibody was purchased from Abcam (Cambridge, MA, USA).

### 2.2. Cells and Virus

Cells A549, HEK293T, MDCK, and HeLa were cultured in Dulbecco’s modified Eagle’s medium (DMEM), SupT1 cells in RPMI1640 (Gibco, Carlsbad, CA, USA) at 37 °C in a 5% CO_2_ incubator. All media were supplemented with 10% fetal bovine serum (FBS; Gibco, Carlsbad, CA, USA). HEK293T-Gluc cells were generated by transfection of plasmid DNA pLenti6-Gluc that constitutively expresses the negative strand RNA of Gluc gene, which is converted into the positive strand upon IAV infection and expresses Gluc enzyme [25]. A549-5Ps cells contain an IAV mini-genome replicon, which was generated via transduction of A549 cells with lentiviral particles carrying PB2, PB1, PA, NP and Gluc/Bsd genes [26]. Influenza A/WSN/33 (H1N1) was generated by transfecting eight plasmids, each coding one IAV RNA fragment, into HEK293T cells, followed by co-culture with MDCK, as described by Hoffmann et al. [24]. A/PR/8/34 (H1N1) were propagated in embryonated chicken eggs according to classical virological techniques. A/Beijing/30/95 (H3N2) was provided kindly by Dr. Wenjie Tan (China CDC). Enterovirus 71 (EV71) strain EV71-HP and VSV-G pseudotyped HIV-1 pNL4-3Luc (R-E-) were described previously [27,28]. ZIKV (strain FSS13025) was a kind gift of Dr. Mark Wainberg (McGill University). In accordance with the rules and regulations for work with viral pathogens in China, all experiments with infectious IAV, EV71 and Zika virus were conducted in a biosafety level 2 facility at the Institute of Medicinal Biotechnology, which were approved by the Institutional Biosafety Committee.

### 2.3. Cell Transfection

For plasmid DNA transfection, cells were transfected with indicated plasmids in a six-well plate using Lipofectamine 2000 (Invitrogen, Carlsbad, CA, USA). Then, cells were cultured for another 24 h for transient expression. esiRNA and siRNA transfection was conducted with the Lipofectamine RNAiMAX reagent (Invitrogen, Carlsbad, CA, USA) according to the manufacturer’s instructions. HEK293T or HEK293T-Gluc cells were reverse-transfected with 20 nM esiRNA or 50 nM siRNA for 48 h, followed by virus infection or plasmid DNA transfection. A549-5Ps cells were transfected twice with esiRNAs at 16 h intervals to achieve maximal transfection efficiency. esiRNA, known as endoribonuclease-prepared siRNA, was produced by employing endonucleolytic cleavage of target specific long double-stranded RNAs by RNase III in vitro. esiRNA is a heterogeneous mixture of siRNAs leading to a more specific and effective knockdown. esiEGFP (EHUEGFP) and esiPAAN (EHNC018541) were purchased from Sigma-Aldrich (St. Louis, MO, USA). A lncRNA Smart Silencer for PAAN containing three siRNA and three antisense oligonucleotides was also purchased from RiboBio Co., Ltd. (Guangzhou, China). The siRNAs target sequences are CCTCTGCTACTGCCTGACA, CCAGGCTGTGATCGTCACA and CCTTTGAAATATCTCAACA; antisense oligonucleotides target sequences are GGAAATGGACCTTGGCAAAC, GAAAGTGGCCCAAGAAGCAC and GGTCCTCA CAGGCCTTCAAC.

### 2.4. IAV Infection and Virus Titer Assay

Cells were incubated with IAV at the indicated MOIs for 1 h at room temperature, and then cultured for 24 h at 37 °C in fresh DMEM. Viral titers were determined on MDCK cells by 50% tissue-culture infectious dose (TCID_50_) using the method described by Reed and Muench [29].

### 2.5. RNA Isolation and Quantitative RT-PCR (qRT-PCR)

Total RNA was extracted from cells using Trizol reagent (Invitrogen, Carlsbad, CA, USA). The cDNA was synthesized using Primescript RT Master Kit (Takara, Tokyo, Japan) with Random and oligo(dT) primers or M-MLV Reverse Transcriptase (Promega, Madison, WI, USA) with specific primers. qRT-PCR was performed using SYBR premix Ex Taq II kit (Takara) according to the manufacturer’s instruction. The primer sets we used include forward primer 5′-CCTGCTGCTGCCTAAGAGAA-3′ and reverse primer 5′-GAAGCGCAGGATGCTTGT-3′ for lncRNA-PAAN; forward primer 5′-GGCTGTTTACCAGACT CCGACA-3′ and reverse primer 5′-CACAAAGCCTGGCAGCTCTCTA-3′ for MxA; forward primer 5′-GTCCACTGGCGTCTTCACCA-3′ and reverse primer 5′-GTGGCAGTGATGGCATGGAC-3′ for GAPDH; reverse transcription primer 5′-GGCCGTCATGGTGGCGAATGAATGGACGGAGAACA AGGATTGC-3′, forward primer 5′-CTCAATATGAGTGCAGACCGTGCT-3′ and reverse primer 5′-GGCCGTCATGGTGGCGAAT-3′ for NP vRNA; reverse transcription primer 5′-CCAGATCGTTC GAGTCGTTTTTTTTTTTTTTTTTCTTTAATTGTC-3′, forward primer 5′-CGATCGTGCCCTCCT TTG-3′ and reverse primer 5′-CCAGATCGTTCGAGT CGT-3′ for NP mRNA. For quantification, the 2^−ΔΔ*C*t^ method was used to calculate the relative RNA levels against GAPDH.

### 2.6. Gluc Acitivity Assay

Gluc activity was measured as described previously by Tannous [30]. Briefly, coelenterazine-h (16.7 μM in phosphate-buffered saline (PBS)) was equilibrated for 30 min in the dark at room temperature. Then, cell culture supernatants were added to the wells in white and opaque 96-well plates, followed by automated injection of 60 μL coelenterazine-h per well. The photon counts for 0.5 s were acquired using Centro XS3 LB 960 microplate luminometer (Berthold Technologies, Bad Wildbad, Germany).

### 2.7. Co-Immunoprecipitation (Co-IP) and Western Blot

For Co-IP, HEK293T cells were collected and lysed in 200 μL buffer containing 25 mM Tris (pH 7.4), 150 mM NaCl, 1% NP-40, 1 mM EDTA, 5% glycerol (Thermo Scientific, Waltham, MA, USA), supplemented with protease inhibitor cocktail (Roche, Basel, Switzerland). The lysates were incubated with anti-PB1 antibody for 4 h and then with protein A/G agarose (Beyotime Biotechnology, Shanghai, China) overnight at 4 °C. The beads were washed with PBS six times, and the samples were analyzed by western blotting.

For western blotting, protein samples were denatured at 100 °C in SDS-PAGE loading buffer for 10 min and separated by SDS-PAGE. Proteins were transferred onto PVDF membranes (Millipore, Burlington, MA, USA) and immunoblotted with indicated antibodies and horseradish peroxidase-conjugated secondary antibodies (Zhongshan Jinqiao Biotechnology, Beijing, China). The membranes were incubated with the Chemiluminescent HRP Substrate (Millipore) and protein signals were recorded using Gel Doc XR+ molecular imager (Bio-Rad, Hercules, CA, USA).

### 2.8. Immunofluorescence (IF)

Cells were washed twice with ice-cold PBS, fixed in 4% paraformaldehyde for 10 min, and permeabilized in 0.25% Triton X-100 for 10 min. After blocking with 1% bovine serum albumin (BSA) for 1 h, cells were incubated with anti-NP antibody (1:1000) overnight at 4 °C, and Alexa-conjugated secondary antibodies (Invitrogen) (1:1000) for 1 h at room temperature. The cells were then stained with DAPI (4′,6′-diamidino-2-phenylindole dihydrochloride) and mounted using Mounting Medium. Fluorescence signals were visualized using the Zeiss LSM 710 confocal microscope (Oberkochen, Germany).

### 2.9. Fluorescence In Situ Hybridization (FISH)

To detect lncRNA-PAAN, FISH was performed using RNAscope Multiplex Fluorescent Reagent Kit v2 (Advanced Cell Diagnostics, Inc., Newark, NJ, USA) according to the manufacturer’s instructions. Briefly, cells were fixed in 4% paraformaldehyde for 30 min, and digested with protease III prior to hybridization with the target RNA-specific probes for 2 h at 40 °C. Preamplifier, amplifier, HRP-labeled oligos and TSA plus Cyanine3 were then hybridized sequentially at 40 °C. The nuclei were stained with DAPI. Images were acquired using the Zeiss LSM 710 confocal microscope.

### 2.10. Cell Viability Assay

HEK293T cells were transfected with esiPAAN or esiEGFP, and cell viability assay was performed 48 h after transfection, using Cell Counting Kit-8 (CCK8) (Beyotime Biotechnology, Shanghai, China).

### 2.11. Subcellular Fractionation

HEK293T cells that were cultured in a six-well plate were washed with PBS and incubated with 200 μL buffer A (10 mM HEPES, pH 7.9, 10 mM KCl, 1.5 mM MgCl_2_, 0.34 M sucrose, 10% glycerol, 1 mM DTT, 0.1% Triton X-100 and protease inhibitor) for 5 min on ice, followed by low speed centrifugation at 4 °C (1500× *g*, 4 min). The supernatant was further clarified by high speed centrifugation (13,000× *g*, 10 min) to remove cell debris and insoluble aggregates and stored as the cytoplasmic fraction. The pellets were washed once with buffer A without 0.1% Triton X-100, then lysed in 200 μL RIPA buffer (50 mM Tris, pH 7.4, 150 mM NaCl, 1% Triton X-100, 1% sodium deoxycholate, 0.1% SDS and protease inhibitor) and stored as nuclear fraction. For RNA quantification in the cytoplasm and nuclei, the subcellular fractions of A549 cells were first prepared in the presence of RNase inhibitor as described above. The levels of lncRNA-PAAN and GAPDH mRNA in cytoplasmic and nuclear fractions were determined by qRT-PCR. The percentage of RNA distribution was the ratio of the amount of RNA in each fraction to the total amount of RNA.

### 2.12. Native RNA Immunoprecipitation (RIP) and Cross-Linked RIP

For native RIP, cells were pelleted at 800× *g* for 5 min and resuspended in lysis buffer (25 mM Tris, pH 7.4, 150 mM NaCl, 1% NP-40, 1 mM EDTA, 5% glycerol) containing RNase inhibitor and protease inhibitor. Dynabeads Protein G (Invitrogen) were incubated with IgG or antibodies against PB1, PB2, PA and NP at room temperature for 2 h, further incubated with cell lysates by rotating at 4 °C overnight. The Dynabeads were washed with PBST (0.02% Tween20 in PBS) five times. The co-precipitated RNA was extracted with Trizol LS (Invitrogen). The levels of lncRNA-PAAN and GAPDH were detected by qRT-PCR.

Cross-linked RIP was performed as previously reported [31]. In brief, the cells were fixed in 1% formaldehyde in PBS for 10 min and lysed by lysis buffer (50 mM HEPES, pH 7.5, 400 mM NaCl, 1 mM EDTA, 1 mM DTT, 0.5% Triton X-100, 10% Glycerol) with sonication (10 pulses for 10 s). Immunoprecipitation was performed overnight at 4 °C using IgG or anti-PA antibody. After washing five times with lysis buffer, the complex was reverse cross-linked at 70 °C, and RNA was extracted with Trizol LS. The levels of lncRNA-PAAN and GAPDH were detected by qRT-PCR.

### 2.13. RNA Pulldown

lncRNA-PAAN RNA was synthesized using the T7 in vitro transcription kit (Thermo Scientific, Waltham, MA, USA) from the PAAN plasmid DNA and labeled with biotin using the RNA 3′ End Desthiobiotinylation kit (Thermo Scientific). RNA pull-downs were performed using the Magnetic RNA-Protein Pull-Down kit (Thermo Scientific) according to the manufacturer’s instructions. In brief, HEK293T cells infected with IAV for 36 h were harvested in lysis buffer (25 mM Tris, pH 7.4, 150 mM NaCl, 1% NP-40, 1 mM EDTA, 5% glycerol). 50 pmol of biotinylated RNA was incubated with streptavidin magnetic beads for 30 min at room temperature. Then cell lysates were incubated with the RNA-bound beads for 60 min at 4 °C with rotation. After three washes, beads-associated proteins were eluted and analyzed by western blot.

### 2.14. Statistical Analysis

Data are presented as mean ± standard deviation (SD) from at least three independent experiments. Statistical analyses were performed using a two-tailed Student’s *t*-test, or one-way ANOVA with Dunnet’s post hoc test for multiple comparisons by using SPSS software (version 25.0. IBM Corp., Armonk, NY, USA) as appropriate. *P* values ≤ 0.05 were considered significant. * denotes *p* ≤ 0.05, ** indicates *p* ≤ 0.01.

## 3. Results

### 3.1. Characterization of the lncRNA-PAAN

To further explore the role of host lncRNAs in IAV replication, we performed a loss-of-function screen using an esiRNA library targeting human lncRNAs (1304 esiRNAs, Sigma). The screen was conducted in 293T-Gluc cells that express Gaussia luciferase (Gluc) upon IAV infection. The cells were first transfected with esiRNAs prior to infection with IAV, followed by measuring the Gluc activity that reflecting the level of IAV infection. Among the top hits in the screen, a human lncRNA, hereafter called lncRNA-PAAN (PA-Associated Noncoding RNA), was further characterized herein.

**Figure 1:**
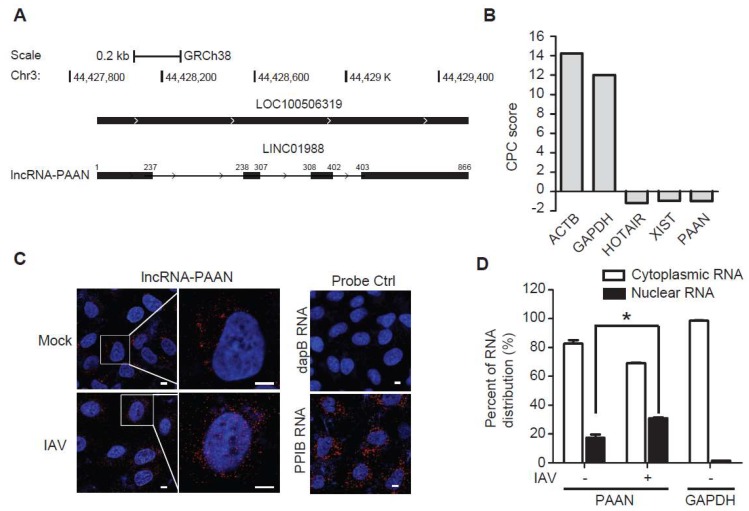
Characterization of lncRNA-PAAN. (**A**) Schematic representation of human lncRNA- PAAN that are transcribed from gene LOC100506319; (**B**) The CPC score of lncRNA-PAAN was calculated using the web-based tool available at cpc.cbi.pku.edu.cn/. ACTB and GAPDH were used as controls for genes with validated protein coding capacity. HOTAIR and Xist were used as controls for validated lncRNA genes. All sequences were scored for their estimated protein-coding capacity. A score <0 indicates non-coding, and a score >0 indicate coding; (**C**) RNA-FISH and confocal imaging to show the localization of lncRNA-PAAN (in red) in A549 cells infected with or without IAV (MOI = 0.5) for 24 h. Localization of the housekeeping gene cyclophilin B (PPIB) RNA was also detected, the results serve as the positive control. A probe specific to bacterial dapB RNA was used as the negative control. Nuclei were stained with DAPI (blue). Scale bar, 5 μm; (**D**) A549 cells were infected with or without IAV (MOI = 0.5) for 24 h. The RNA levels of lncRNA-PAAN, GAPDH were determined by qRT-PCR in cytoplasmic and nuclear fractions. The percentages of distribution of PAAN and GAPDH in cytoplasm and nucleus were shown. IAV means the WSN strain unless otherwise specified. +: IAV infection; −: Mock; *: *p* ≤ 0.05.

The human *lncRNA-PAAN* gene (LOC100506319) is located on chromosome 3p21.31, intergenic to the two coding genes *TCAIM* and *ZNF445*, with distances of 15 kb and 10 kb, respectively. LncRNA-PAAN is encoded by 4 exons (866 nt, https://www.ncbi.nlm.nih.gov/gene/? term = LINC01988) (Figure 1A). We first analyzed the theoretical coding potential of both isoforms using the Coding Potential Calculator (CPC) program. Results in Figure 1B show that the CPC score for lncRNA-PAAN is −0.980, which is in a range similar to that obtained for the validated lncRNA HOTAIR (−1.187) and XIST (−0.945). In contrast, the CPC scores for the coding genes ACTB (actin beta) and GAPDH are 14.242 and 12.007, respectively. These data suggest that lncRNA-PAAN lacks coding potential. qRT-PCR quantification analysis showed 29 ± 6.2 molecules per cell of lncRNA-PAAN in HEK293T cells. Next, we determined the subcellular localization of lncRNA-PAAN by performing RNA fluorescence in situ hybridization (FISH) and fractionation analysis. The results showed that lncRNA-PAAN was primarily localized in the cytoplasm in both IAV-infected and uninfected cells, while IAV infection resulted in an approximate 60% increase in the proportion in the nuclei to total lncRNA-PAAN (Figure 1C,D). Together, these results suggested that lncRNA-PAAN was predominantly localized in the cytoplasm and IAV infection promoted its nuclear localization.

### 3.2. lncRNA-PAAN is Induced by IAV Infection Independent of Interferon

**Figure 2:**
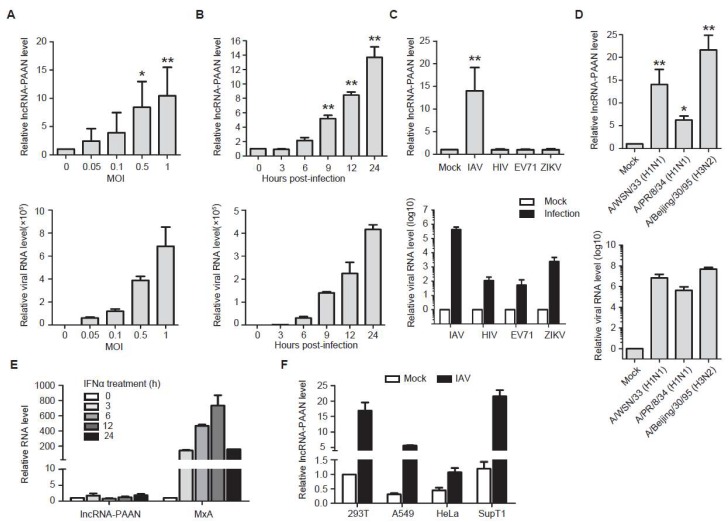
LncRNA-PAAN is induced by IAV infection independent of interferon. (**A**) HEK293T cells were infected with IAV at the indicated MOIs for 24 h. Levels of lncRNA-PAAN (upper panel) and viral RNA (lower panel) were determined by qRT-PCR; (**B**) HEK293T cells were infected with IAV (MOI = 1) for indicated time intervals. Levels of lncRNA-PAAN (upper panel) and viral RNA (lower panel) were determined by qRT-PCR; (**C**) HEK293T cells were infected with IAV, HIV-1, EV71 and ZIKV (MOI = 1) for 24 h. Levels of lncRNA-PAAN (upper panel) and viral RNA (lower panel) were determined by qRT-PCR; (**D**) HEK293T cells were infected with A/WSN/33(H1N1), A/PR/8/34(H1N1) and A/Beijing/30/95(H3N2) (MOI = 1) for 24 h. Levels of lncRNA-PAAN (upper panel) and viral RNA (lower panel) were determined by qRT-PCR; (**E**) Levels of lncRNA-PAAN and MxA mRNA in A549 cells that were treated with IFN-α (1000 IU/mL) for the indicated time intervals; (**F**) Levels of lncRNA-PAAN in different human cell lines after IAV infection (MOI = 1) for 24 h. Levels of lncRNA-PAAN were quantified by qRT-PCR; In (**A**–**F**) data, the level of GAPDH mRNA was also determined and used as the internal control to normalize the level of lncRNA-PAAN. The value of the control group was arbitrarily set as 1. IAV means the WSN strain unless otherwise specified. *: *p* ≤ 0.05; **: *p* ≤ 0.01.

We next examined whether IAV infection affects the expression of lncRNA-PAAN. To this end, HEK293T cells were infected with IAV of different MOIs. The levels of lncRNA-PAAN were determined by qRT-PCR. The results showed that both lncRNA-PAAN (Figure 2A, upper panel) and viral RNA (Figure 2A, lower panel) were upregulated in an IAV dose-dependent manner, with 10-fold increase at the highest MOI infection. Moreover, results of time-course IAV infections showed that lncRNA-PAAN expression already increased at 3 h post-infection, and its level continued to rise with the progression of IAV infection (Figure 2B). Besides influenza A/WSN/33 virus shown in Figure 2A,B, the infections of other IAV stains including A/Beijing/30/95 (H3N2) isolate and A/PR/8/34 (H1N1) also significantly induced the expression of lncRNA-PAAN (Figure 2D). However, the level of lncRNA-PAAN did not change upon infection of VSV-G pseudotyped HIV-1, EV71 or Zika virus (Figure 2C, upper panel), whose replications were determined by qRT-PCR quantification of viral RNA (Figure 2C, lower panel). In addition, IFN-α did not affect lncRNA-PAAN expression, although MxA expression was drastically stimulated (Figure 2E). We further measured the relative abundance of lncRNA-PAAN in several human cell lines, including HEK293T, SupT1 and HeLa cells and observed lncRNA-PAAN expression in all these cell lines, with higher levels in HEK293T and SupT1 cells. Importantly, lncRNA-PAAN expression was profoundly elevated by IAV infection in HEK293T, A549 and SupT1 cells, with moderate increase in HeLa cells which are less permissive to IAV infection [32] (Figure 2F). Together, these data together demonstrate that IAV infection specifically up-regulates the expression of lncRNA-PAAN.

### 3.3. Downregulation of lncRNA-PAAN Inhibits IAV Replication

**Figure 3:**
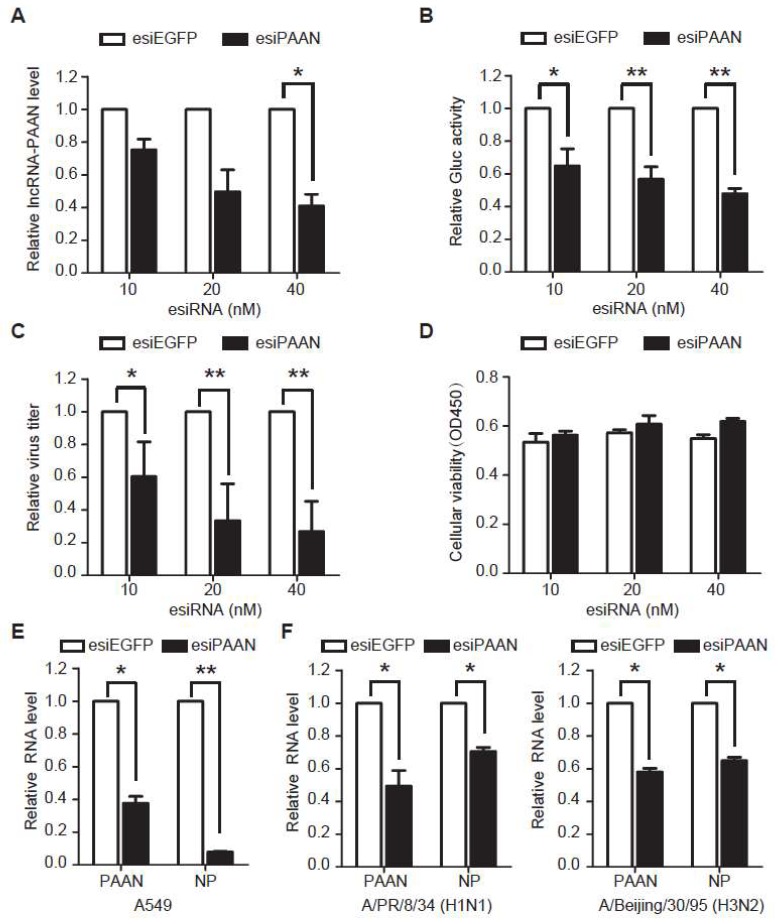

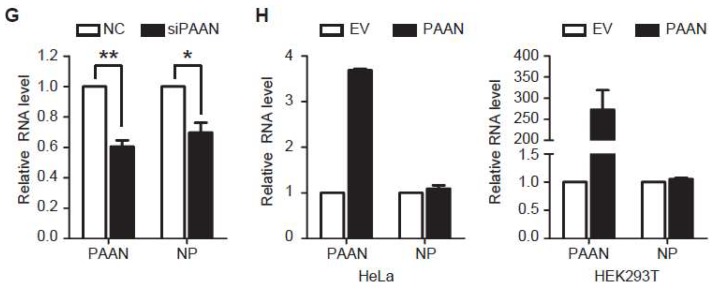
Downregulation of lncRNA-PAAN inhibits IAV replication. (**A**–**C**) HEK293T-Gluc cells were transfected with the indicated amounts of either esiPAAN or control esiEGFP for 48 h, followed by infection with IAV (MOI = 0.5) for 24 h. Levels of lncRNA-PAAN were quantified by qRT-PCR and GAPDH mRNA levels were used to normalize the levels of lncRNA-PAAN (**A**). Viral infectivity was determined by measuring Gluc activity (**B**) and infectious titers of progeny IAV in supernatants were determined by performing the TCID50 assay (shown in (**C**)); (**A**–**C**) data are normalized to control group, with the value of control arbitrarily set as 1; (**D**) HEK293T cells were transfected with the indicated amounts of esiPAAN or esiEGFP for 48 h. Cell viability was determined by performing the CCK8 assay; (**E**) A549 cells were transfected with either esiPAAN or control esiEGFP (40 nM) for 24 h, followed by infection with IAV (MOI = 0.5) for 36 h. Levels of lncRNA-PAAN and viral RNA were determined by qRT-PCR; (**F**) HEK293T cells were transfected with either esiPAAN or control esiEGFP (20 nM) for 36 h, followed by infection with A/PR/8/34(H1N1) or A/Beijing/30/95(H3N2) (MOI = 0.5) for 24 h. Levels of PAAN RNA and viral RNA were determined by qRT-PCR; (**G**) HEK293T cells were transfected with either siPAAN or negative control siRNA (50 nM) for 36 h, followed by infection with IAV (MOI = 0.5) for 24 h. Levels of PAAN RNA and viral RNA were determined by qRT-PCR; (**H**) HeLa and HEK293T cells were transfected with plasmid DNA expressing lncRNA-PAAN or empty vector (EV) for 24 h and infected with IAV (MOI = 0.5) for 36 h. The levels of lncRNA-PAAN and viral RNA were quantified by qRT-PCR; In (**E**–**H**) data, the level of GAPDH mRNA was also determined and used as the internal control to normalize the level of lncRNA-PAAN, and results are normalized to the values of the control, with the value of the control arbitrarily set as 1. IAV means the WSN strain unless otherwise specified. *: *p* ≤ 0.05; **: *p* ≤ 0.01.

To investigate the role of lncRNA-PAAN in IAV replication, we transfected HEK293T-Gluc cells with esiPAAN targeting lncRNA-PAAN or esiEGFP as a control, followed by IAV infection. HEK293T-Gluc cells express Gaussia luciferase (Gluc) upon IAV infection and thus Gluc activity in the supernatant reports the level of IAV infection. The results of qRT-PCR showed gradual decline of lncRNA-PAAN expression with increasing amounts of esiPAAN transfected (Figure 3A). As a result, Gluc activity and titers of progeny IAV decreased in an esiPAAN dose-dependent manner. In addition, results of the cytotoxicity assays showed that neither esiPAAN transfection nor esiEGFP transfection affected cell viability (Figure 3D), demonstrating that inhibition of IAV replication by lncRNA-PAAN silencing is not a result of cell toxicity. It should be noted that GAPDH, which we used as a normalization control in RNA quantification, was reported to decrease due to host shutoff during IAV infection, suggesting a likely underestimation of lncRNA knock-down. Furthermore, we found that in A549 cells transfected with esiPAAN, viral RNA level was reduced by approximate 90% compared to that in the control cells (Figure 3E). Similarly, a marked reduction in viral replication was observed for other IAV stains including A/Beijing/30/95 (H3N2) isolate and A/PR/8/34 (H1N1) in lncRNA-PAAN silencing cells (Figure 3F).

To exclude the possibility of off-target effects of esiRNA, we have examined the effect of lncRNA-PAAN knockdown by specific siRNA on IAV replication. The result showed a significant reduction in viral RNA levels in the cells transfected with siRNA specific for lncRNA-PAAN compared to that in scramble control siRNA group (Figure 3G). Taken together, these data demonstrate that lncRNA-PAAN is required for efficient IAV replication.

In addition, we have further tested whether ectopically expressed lncRNA-PAAN promotes IAV replication. HeLa and HEK293T cells were transfected with the DNA constructs expressing lncRNA-PAAN, resulting in a significant increase in the lncRNA levels. However, no effect on viral replication in both cell lines was observed (Figure 3H). One possible explanation for this observation is that the increased lncRNA-PAAN induced by IAV infection may be enough for warranting efficient viral replication.

### 3.4. LncRNA-PAAN Knockdown Reduces Viral RNA Transcription and Replication

**Figure 4:**
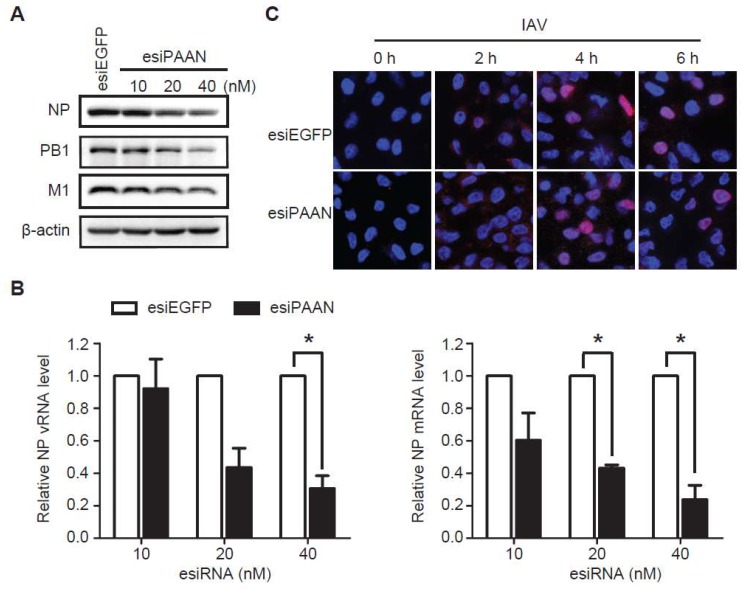
LncRNA-PAAN knockdown reduces viral RNA transcription and replication. (**A**,**B**) HEK293T cells were transfected with esiPAAN or esiEGFP, followed by infection with IAV (MOI = 0.5) for 24 h. Levels of viral NP, PB1 and M1 proteins were analyzed by western blot (**A**). Levels of vRNA and mRNA of the NP gene were determined by qRT-PCR. GAPDH mRNA was used as the internal control, and data are normalized to the control group with the value of control arbitrarily set as 1 (**B**). (**C**) HEK293T cells were transfected with esiPAAN or esiEGFP, then exposed to IAV infection (MOI = 3). At the indicated time points, cells were stained for nuclei (blue) and NP (red) in the indirect immunofluorescence assay. IAV means the WSN strain unless otherwise specified. *: *p* ≤ 0.05.

Next, we analyzed the effect of lncRNA-PAAN silencing on the expression of viral proteins and viral RNA in IAV infected cells. The results of western blots showed that the levels of viral protein NP, PB1 and M1 gradually declined with increasing amounts of esiPAAN transfected (Figure 4A). We also observed that levels of both vRNA and mRNA of NP reduced by approximately 70% in the lncRNA-PAAN knockdown cells, compared to that in the control cells (Figure 4B). These data suggest that lncRNA-PAAN is required for the expression of viral RNA and viral proteins. In addition, we examined the effect of lncRNA-PAAN knockdown on IAV entry and post-entry by monitoring the nuclear entry of viral RNP, and observed no significant change (Figure 4C), which further supports the role of lncRNA-PAAN in the later stage of IAV infection including viral RNA transcription and replication.

### 3.5. LncRNA-PAAN Knockdown Impairs IAV Polymerase Activity and the PB1-PB2 Interaction

**Figure 5:**
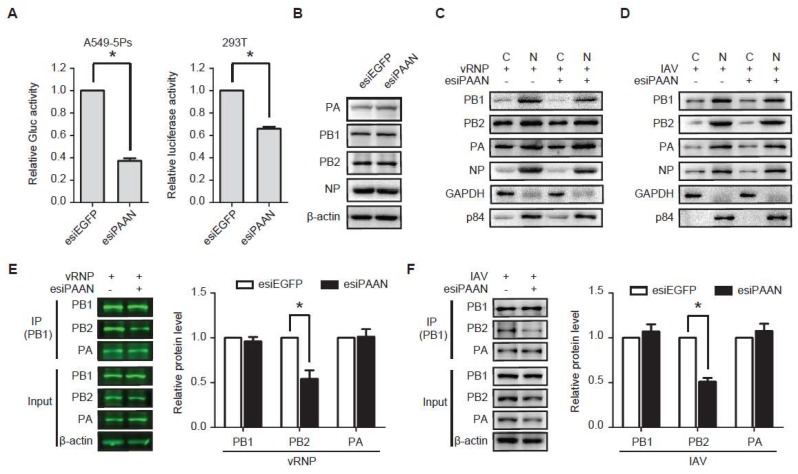
LncRNA-PAAN knockdown impairs PB1-PB2 association and the RdRp activity. (**A**) Gluc activities were determined in A549-5Ps cells that were transfected with esiPAAN or esiEGFP (left panel). Luciferase activities were measured in HEK293T cells that were transfected with esiPAAN or esiEGFP, followed by co-transfection of RNP reconstitution plasmids (PB1, PB2, PA, NP) and the reporter plasmid used to transcribe an IAV-like RNA (right panel); (**B**) A549-5Ps cells were transfected with esiPAAN or esiEGFP. Levels of viral PA, PB1, PB2 and NP protein were determined by western blotting; (**C**,**D**) HEK293T cells were transfected with esiEGFP or esiPAAN, followed by co-transfection of plasmid DNA encoding vRNP subunits (PB1, PB2, PA, NP and PolI-luc) (**C**), or infection with IAV (MOI = 0.5) for 24 h (**D**). Levels of viral PA, PB1, PB2 and NP in cytoplasm and nuclei were analyzed by western blotting. The GAPDH protein was used as a cytoplasmic marker, and p84 protein as a nuclear marker. (**E** and **F**) HEK293T cells were transfected with esiEGFP or esiPAAN, followed by co-transfection of plasmid DNA encoding vRNP subunits (**E**), or infection with IAV (MOI = 0.5) for 24 h (**F**). Cell lysates were immunoprecipitated with anti-PB1 antibody. The inputs and the PB1-immunoprecipitated samples were analyzed by western blotting with the indicated antibodies. The intensity of protein band was determined using the Odyssey infrared imaging system (**E**) and Image J program (**F**). Right panel represents relative levels of PB1, PB2 and PA in the immunoprecipitated samples, which were calculated by normalization to control groups. IAV means the WSN strain unless otherwise specified. *: *p* ≤ 0.05.

We further examined the effect of lncRNA-PAAN knockdown on viral RNA transcription and replication in an IAV mini-genome replicon system that was stably engineered in the A549-5Ps cells. In this cell line, IAV polymerase activity was reconstituted by stably expressing RdRp subunits (PA, PB1 and PB2), NP and a vRNA-like reporter gene Gluc. Results showed that lncRNA-PAAN knockdown reduced Gluc level by 60% (Figure 5A, left panel), suggesting an inhibition of IAV RNA replication. A similar effect of lncRNA-PAAN knockdown on the RdRp activity was observed in HEK293T cells transfected with plasmid DNA encoding vRNP subunits (PB1, PB2, PA, NP and PolI-luc) (Figure 5A, left panel). Results of western blotting showed that silencing lncRNA-PAAN did not change the levels of PA, PB1, PB2 and NP in A549-5Ps cells (Figure 5B) and in transfected HEK293T cells (Figure 5E). Furthermore, similar subcellular distributions of these viral proteins were observed in cells that were transfected with either esiPAAN or control esiRNA, suggesting that lncRNA-PAAN is not required for nuclear import of the RdRp complex in replicon-expressing cells (Figure 5C) and IAV-infected cells (Figure 5D).

The IAV RdRp complex consists of PB1, PB2 and PA that are all essential for the replication and transcription of viral genome. Thus, we tested whether lncRNA-PAAN knockdown affects the formation of IAV RdRp complex in cells transiently expressing vRNP or IAV infected cells by performing the Co-IP assay using the anti-PB1 antibody. The results showed that approximate 50% less PB2 protein was co-immunoprecipitated with PB1 in the esiPAAN-transfected cells compared with that in the esiEGFP-transfected control cells, whereas esiPAAN did not affect the association of PA with PB1 (Figure 5E). Similarly, the inhibitory effect of lncRNA-PAAN knockdown on the interaction of PB1 and PB2 was observed in the IAV-infected cells (Figure 5F). These data suggest a role of lncRNA-PAAN in the association of PB1 with PB2.

### 3.6. LncRNA-PAAN Interacts with IAV PA

**Figure 6:**
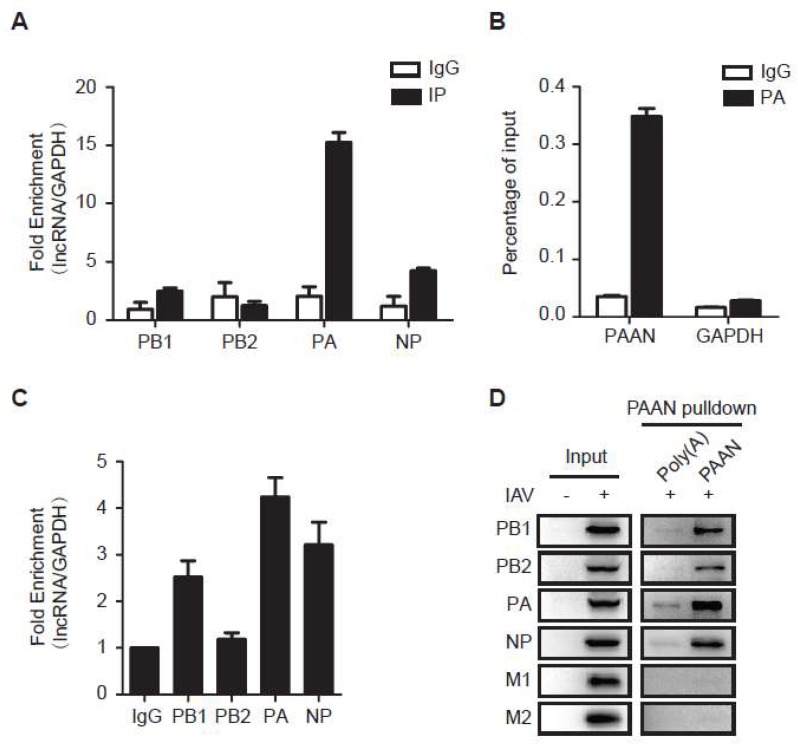
LncRNA-PAAN is associated with IAV PA protein. (**A**,**C**) RIP experiment was performed to detect the association of lncRNA-PAAN RNA with the viral proteins. HEK293T cells were transfected with plasmid DNA expressing PB1, PB2, PA or NP (**A**) and infected with IAV (MOI = 0.5) for 36 h (**C**). Cell lysates were immunoprecipitated with the indicated antibodies, and levels of lncRNA-PAAN and GAPDH were determined by qRT-PCR. IgG was used in immunoprecipitation; the results serve as the control; (**B**) Cross-linked RIP experiment was performed to detect the association of lncRNA-PAAN with viral PA protein. HEK293T cells were transfected with plasmid DNA expressing lncRNA-PAAN and PA. Cells was crosslinked, and lysates were immunoprecipitated with the anti-PA antibody. Levels of lncRNA-PAAN and GAPDH were determined by qRT-PCR. IgG was used in immunoprecipitation; the results serve as the control; (**D**) RNA pulldown assay was performed to detect the association of lncRNA-PAAN RNA with the viral proteins. HEK293T cells were infected with IAV (MOI = 0.5) for 36 h. Cell lysates were incubated with biotinylated RNA probes of PAAN. The pull-down products were examined by western blotting with the indicated antibodies. IAV means the WSN strain unless otherwise specified.

Studies have shown that lncRNAs interact with proteins and regulate protein localization and stability [33,34,35]. We therefore performed native RNA immunoprecipitation (RIP) assay to examine whether lncRNA-PAAN is associated with the RdRp complex. PB1, PB2, PA or NP was individually expressed in cells, then immunoprecipitated. Levels of lncRNA-PAAN that was co-immunoprecipitated were determined by qRT-PCR. The enrichment of lncRNA-PAAN in each precipitated sample was calculated against the level of lncRNA-PAAN in the input, and the ratio was further normalized with that of control GAPDH mRNA in the same sample, to show selective association with lncRNA-PAAN. The results showed 15-fold enrichment of lncRNA-PAAN in the PA-immunoprecipitated sample, and moderate enrichment with PB1 and NP (Figure 6A), suggesting preferable association of lncRNA-PAAN with IAV PA protein. In agreement with our conclusion, the result of cross-linked RNA Immunoprecipitation (CL-RIP) showed significant enrichment of lncRNA-PAAN compared to control GAPDH mRNA in the PA-immunoprecipitated sample (Figure 6B).

In addition, the association of lncRNA-PAAN and IAV proteins in IAV-infected cells was assessed using RIP assay. The result showed the most significant enrichment of lncRNA-PAAN in the PA-immunoprecipitated sample in IAV-infected cells, and relative less enrichment of lncRNA-PAAN with PB1 and NP (Figure 6C). We further validated the interaction between lncRNA-PAAN and IAV proteins by performing RNA pull-down experiments using IAV-infected cell lysates. After incubating biotinylated lncRNA-PAAN probe with whole cell extracts, the RNA pulldown samples were analyzed by western blotting with the indicated antibodies. As shown in Figure 6D, the components of the viral RNP complex including PA, PB1, PB2 and NP, not M1 and M2 protein, was specifically precipitated with lncRNA-PAAN. Notably, PA is the most abundant one found in the RNA pull-down samples, and the enrichment of viral proteins co-precipitated with lncRNA-PAAN is similar to what we found in RIP analysis. Taken together, these data suggest preferable association of lncRNA-PAAN with IAV PA protein.

## 4. Discussion

RNAi-based genome-wide screens and proteomic studies have been performed to identify cellular proteins that might associate with IAV and modulate IAV replication [36]. Among these host proteins, fragile X mental retardation protein (FMRP) targets IAV RNA synthesis machinery and facilitates virus replication by stimulating RNP assembly [37]. IAV recruits host protein kinase C to control assembly and activity of its replication machinery [38]. In this work, we have discovered a new lncRNA, named lncRNA-PAAN that is specifically induced by IAV infection in an interferon independent manner. Our data further demonstrate that lncRNA-PAAN acts as a positive regulator of influenza virus replication by promoting the assembly of RdRp complex and thereby enhancing viral RNA polymerase activity. Therefore, our study provides an example of host lncRNA that, as with host proteins, acts as a co-factor of IAV by assisting viral RNA synthesis.

We observed that expression of lncRNA-PAAN was significantly augmented upon infection of influenza virus, but not VSV-G pseudotyped HIV-1, EV71, nor ZIKV (Figure 2C). This indicates that lncRNA-PAAN is specifically hijacked by influenza A virus to support its replication. In agreement with this interpretation, IAV was found to increase lncRNA-PAAN expression in different cell types, albeit to various degrees (Figure 2). Interestingly, there appears to exist a correlation between the degree of lncRNA-PAAN enhancement and the level of IAV replication in a specific cell line. Level of lncRNA-PAAN increased profoundly at 3 h post-infection when viral proteins and RNA started to express in IAV infected cells, and then continued to increase albeit at a lower rate (Figure 2B). This kinetics of increase suggests a possible synchronization of lncRNA-PAAN synthesis and IAV replication. The detailed mechanism awaits further investigation.

Transcription and replication of IAV genome by the RdRp complex occur in the nucleus. IAV RdRp consists of PB1, PB2 and PA proteins that are synthesized in the cytoplasm, followed by transportation into the nucleus to assemble vRNP. Studies on the assembly pathway and nuclear import of the polymerase complex suggest that the PB1 and PA subunits interact in the cytoplasm and are subsequently transported as a dimer into the nucleus that is likely mediated by the importin RanBP5 [39,40]. In contrast to PA-PB1 with RanBP5, PB2 would independently enter the nucleus as a monomer, probably through the importin-α pathway [41], and then bind to the PB1-PA dimer to assemble a functional complex (PA-PB1-PB2). Since lncRNA-PAAN silencing reduces the level of PB2 subunit in the RdRp complex, without affecting the expression of the RdRp subunits nor their nuclear import (Figure 5), we concluded that lncRNA-PAAN promotes the assembly of RdRp complex, thus contributing to IAV replication.

The X-ray crystallography structures of the complete RdRp complex showed that the PB1 subunit is positioned at the center of the polymerase complex, with its N- and C-terminal extensions facilitating interactions with the PA and PB2 subunits, respectively [42]. Given that our data showed a strong association of lncRNA-PAAN with PA protein but not PB2 protein (Figure 6), lncRNA-PAAN most likely facilitates PB2 and PB1 in an indirect manner. One possibility might be that PB2 preferably binds to the PA-PB1 dimer that is associated with lncRNA-PAAN, to efficiently form the RdRp complex. A recent work suggests that RanBP5 disassociation from the PA-PB1 dimer is coupled to assembly of the polymerase with viral promoter RNA [43], therefore it is also possible that lncRNA-PAAN regulates the disassociation process, thereby indirectly promoting the RdRp complex assembly. In addition, binding of lncRNA-PAAN to PA may enhance an interaction between PA and PB2, which was reported previously with unknown function [44]. In any event, further experiments are required to elucidate how lncRNA-PAAN regulates the assembly of the IAV polymerase complex. Especially, imaging the RdRp assembly in living cell during overexpression of lncRNA-PAAN and esiPAAN treatment as well as the co-localization of lncRNA-PAAN and the RdRp component shall provide direct evidence for understanding the cellular dynamics and function of lncRNA-PAAN.

In summary, we have discovered lncRNA-PAAN as a new host factor that is exploited by IAV to promote viral replication. Our findings have further illuminated the important roles of lncRNAs in viral replication and may inspire the development of new antiviral therapies.
